# Tissue-specific Calibration of Real-time PCR Facilitates Absolute Quantification of Plasmid DNA in Biodistribution Studies

**DOI:** 10.1038/mtna.2016.79

**Published:** 2016-10-04

**Authors:** Joan K Ho, Paul J White, Colin W Pouton

**Affiliations:** 1 Drug Discovery Biology, Monash Institute of Pharmaceutical Sciences, Monash University (Parkville Campus), Melbourne, Australia; 2 Drug Delivery, Disposition and Dynamics, Monash Institute of Pharmaceutical Sciences, Monash University (Parkville Campus), Melbourne, Australia.

## Abstract

Analysis of the tissue distribution of plasmid DNA after administration of nonviral gene delivery systems is best accomplished using quantitative real-time polymerase chain reaction (qPCR), although published strategies do not allow determination of the absolute mass of plasmid delivered to different tissues. Generally, data is expressed as the mass of plasmid relative to the mass of genomic DNA (gDNA) in the sample. This strategy is adequate for comparisons of efficiency of delivery to a single site but it does not allow direct comparison of delivery to multiple tissues, as the mass of gDNA extracted per unit mass of each tissue is different. We show here that by constructing qPCR standard curves for each tissue it is possible to determine the dose of intact plasmid remaining in each tissue, which is a more useful parameter when comparing the fates of different formulations of DNA. We exemplify the use of this tissue-specific qPCR method by comparing the delivery of naked DNA, cationic DNA complexes, and neutral PEGylated DNA complexes after intramuscular injection. Generally, larger masses of intact plasmid were present 24 hours after injection of DNA complexes, and neutral complexes resulted in delivery of a larger mass of intact plasmid to the spleen.

## Introduction

Determining the tissue distribution profile of DNA plasmid after parenteral administration is an important tool in designing DNA-based therapies, as it can assist with assessing the likely sites of gene expression, the duration of action, and the degree to which these factors are influenced by the design of the DNA delivery system. Indeed, it has been well established that the composition, size, and surface charge of a complex can govern its distribution to different tissues *in vivo.*^1,2,3,4^ More subtle differences, such as the chain length of the DNA-condensing polymer, can also influence the distribution of the complex to different organs in mice, and in turn, affect transfection efficiency.^5,6^ Accurate determination of the absolute mass of plasmid distributed to different tissue sites is critical to studies of the mechanisms of action of gene delivery systems and understanding the influence of the physicochemical properties of DNA complexes on their biological activity.

Although many studies have used fluorescent or radio-labeling methods to track DNA after administration *in vivo*, these methods do not determine how much of the labeling moiety has been cleaved and separated from the DNA, and therefore cannot differentiate between DNA that is intact and DNA that has been partially degraded.^1^ Thus, studies using such labels cannot establish the distribution profile of active DNA complex to different tissues *in vivo*, but may instead reflect the accumulation or clearance pathway of the degraded products in those given tissues. This limits the value of such pharmacokinetic studies as the true effect of size or surface modifications of DNA complexes on biodistribution cannot be determined. In contrast, real-time quantitative polymerase chain reaction (qPCR) is a sensitive technique than can be used to detect low levels of DNA in tissues.^5,7,8,9,10^ A major advantage of this technique over labeling methods is that qPCR can be targeted such that degraded DNA is not amplified, allowing quantitative determination of intact DNA encoding the gene or expression cassette of interest.

Published qPCR strategies do not allow comparison of the absolute mass of plasmid DNA delivered to different tissues. The amount of target plasmid detected is usually expressed as the concentration of plasmid relative to the amount of genomic DNA (gDNA) in the sample.^8,11^ Although this strategy is adequate for comparing the amount of plasmid accumulated at the *same tissue site* after administration of different gene-based formulations, it does not allow direct comparison of the amount of plasmid accumulated at *different tissue sites*. For example, if 10 ng of plasmid per ng of gDNA is detected in the muscle, and 1 ng of target plasmid per ng of gDNA is detected in the lymph node, it is not possible to conclude that the concentration of DNA in the muscle is 10 times higher than in the lymph node. This is because the efficiency of gDNA extraction from each tissue is unknown. In this study, we present qPCR assay methods that allow the absolute amount of plasmid DNA distributed to different tissues to be determined, and we exemplify the method by studying the difference in biodistribution of naked DNA, cationic and PEGylated complexes.

The use of amphiphilic peptides or lipopeptides (LP) to deliver DNA to cells has become a more common approach in recent years due to the opportunity to include amino acid residues that may assist in overcoming DNA delivery hurdles (*e.g.*, use of histidine residues to enhance endosomal escape).^12,13,14,15^ In this study, we explored the effect of the cationic LP “stearoylCH_2_K_3_” as a DNA carrier in the distribution of the plasmid to different tissues *in vivo*. The design of the LP is based on previous *in vitro* work carried out by our group.^12^ Lysine residues are included to accomplish the condensation and cell uptake of DNA;^16,17,18,19^ histidines, to assist in the endosomal escape;^19,20,21,22^ and cysteine, to assist in the stabilization of the DNA complex by formation of disulphide bonds.^23,24,25,26^ Furthermore, as cationic complexes have been shown to have restricted movement from the site of injection *in vivo*,^1,2,3,27^ a second formulation in which the surface charge of the lipopeptide/DNA (LP/DNA) complex is shielded with a hydrophilic polyethylene glycol (PEG) layer was also assessed. The PEG mantle was provided by incorporation of distearoyl-phosphoethanolamine-(polyethylene glycol_2000_) (DSPE-PEG_2000_) which had the added advantage of neutralizing excess cationic charge.

## Results

### Characterization of DNA complexes

The LP/DNA complexes had a Z-average particle size of 112.5 ± 17.0 nm, a polydispersity index (PI) of 0.19 ± 0.02 and a zeta potential (ZP) of +34.2 ± 1.2 mV. The DSPE-PEG_2000_/LP/DNA complexes had a Z-average of 178.6 ± 49.5 nm, PI of 0.23 ± 0.05 and a ZP of +0.76 ± 0.2 mV.

### Specificity, reproducibility, and validation of the qPCR assay

**Figure 1:**
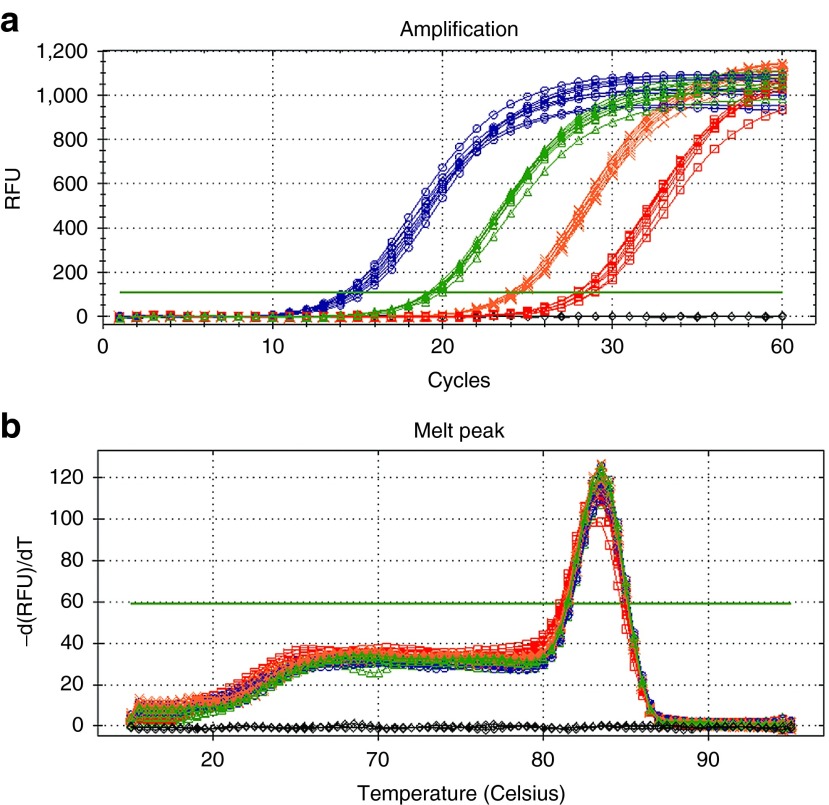
**Typical real-time PCR data obtained in the presence of homogenized tissue.** (**a**) Representative Cq amplification plot of DNA introduced as a standard into the excised muscle tissue plotted against the relative fluorescent units (RFU) and (**b**) the corresponding melting curve.

**Figure 2:**
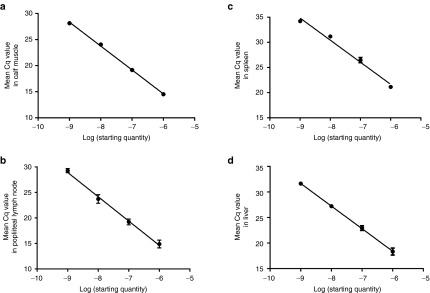
**Standardization of PCR data.** Constructed standard plots of the Cq values versus mass of plasmid DNA introduced into the calf muscle (**a**), popliteal lymph node (**b**), spleen (**c**) and liver (**d**) homogenates. *n* = 3 tissues for each data point. Data is presented as mean ± SEM.

We used a ~9.9 kb luciferase expression plasmid (pCMV-luc) to develop the assay methods. qPCR reactions were designed to amplify a 114 bp fragment including part of the cytomegalovirus (CMV) promoter and part of the luciferase cDNA. The specificity of the reaction was confirmed by the presence of a single peak at ~82.5 °C in the melting curve plots for each sample (**Figure 1**), and a single amplicon product identified by agarose gel electrophoresis (data not shown). The qPCR amplification profile was initially studied by spiking excised tissues with the pCMV-luc plasmid. This revealed consistent quantification cycle (Cq) values for each replicate (**Figure 1**). A low coefficient of variation (CoVar) of the qPCR assay was observed for all tissues spiked with 100 ng of pCMV-luc. CoVars were 0.6% for the calf muscle, 5.2% for the draining lymph node, 3.3% for the spleen, and 3.5% for the liver. This data indicated high reproducibility of the assay with each of the tissue tested. Standard plots of Cq versus log mass of DNA had similar slopes for each tissue, ranging from −4.4 to −4.8, indicating similar amplification efficiencies (62–70%) (**Figure 2**). Linear regression of the plots generated an *R*^2^ value of 0.97 for the draining lymph node and ≥ 0.98 for the other tissues, indicating linearity over the range investigated.

### Comparison of qPCR methods

**Figure 3:**
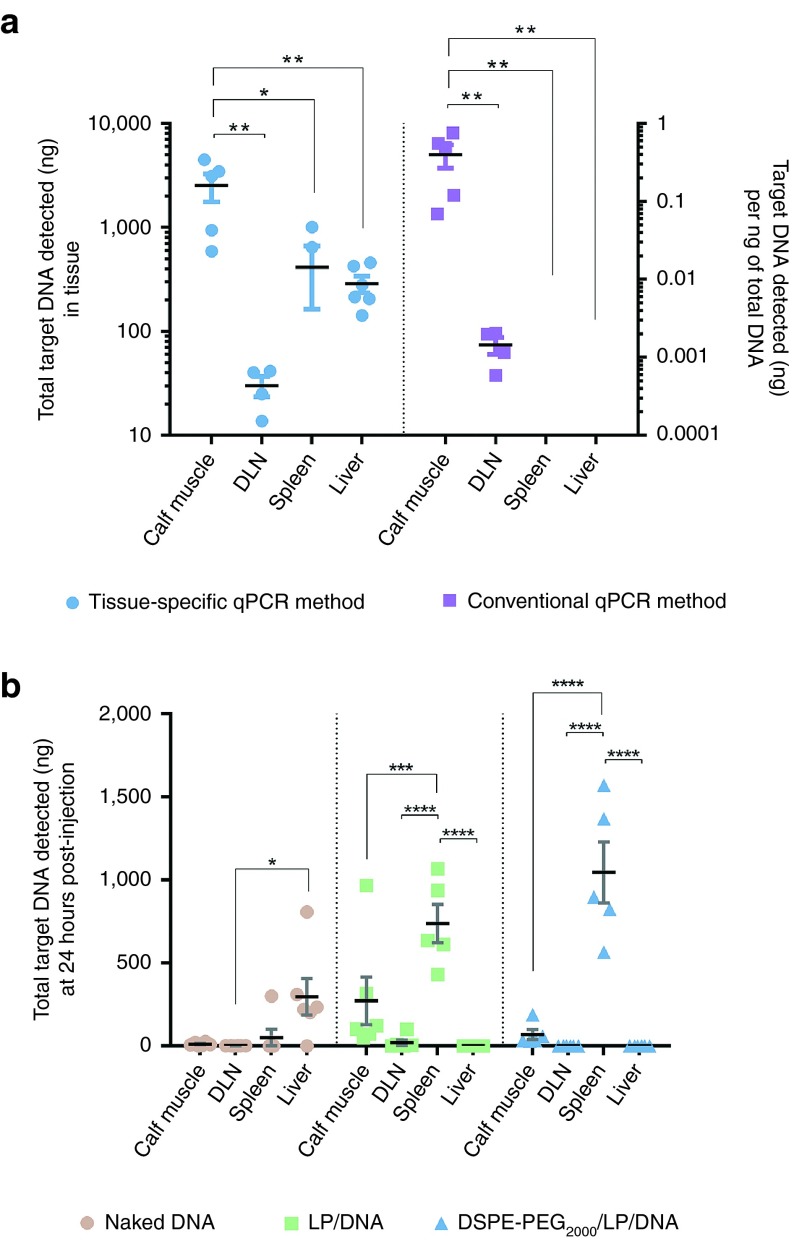
**Use of tissue-specific PCR in biodistribution studies. **(**a**) Tissue distribution of the administered plasmid DNA at 30 minutes post-intramuscular injection into the calf muscle showing the absolute amount (ng) of plasmid in the tissue using our tissue-specific qPCR assay and the amount (ng) of plasmid detected in the tissue per ng of total DNA extracted via conventional qPCR analysis. (**b**) Tissue distribution of the administered plasmid DNA, LP/DNA and DSPE-PEG_2000_/LP/DNA complexes at 24 hours postinjection into the calf muscle using our qPCR method.*P < 0.05, **P < 0.01, ***P < 0.001, ****P < 0.0001. n = 4 – 6 mice for each tissue. Data is represented as mean ± SEM. Values less than the reliable quantification limits of the qPCR assay are plotted as 0.

Analysis of the mass of target DNA detected at each tissue site using our tissue-specific qPCR method and the conventional qPCR method (see “Application of qPCR assay to investigate the biodistribution of plasmid DNA” Methods section) were carried out using mouse tissues 30 minutes after injection of naked plasmid DNA into the calf muscle. Using the tissue-specific qPCR method, the intact target DNA could be detected in all the tissues assayed at this time point (**Figure 3a**). The masses detected in the tissues ranged across three orders of magnitude (ng to µg of plasmid DNA). After 30 minutes, the largest mass of the original dose was found in the calf muscle (5.04%; *n* = 4–6, *P* < 0.05 when compared to the spleen, *P* < 0.01 compared to other tissues). The spleen contained the second highest mass of intact plasmid with a mean of 0.83% of the dose detected (*n* = 4). The draining popliteal lymph node and liver contained 0.06 and 0.58% of the administered dose, respectively. The differences in the amounts of plasmid detected in spleen, lymph node and liver were not statistically significant. Analysis of the same samples using the conventional qPCR method, suggested that at 30 minutes postinjection, the calf muscle contained a higher amount of the plasmid, per ng of total DNA extracted, than the other tissues (0.40 ng; *n* = 4–6, *P* < 0.01). This method suggested that the mass of plasmid detected per ng of total DNA was higher in the draining lymph node than both the spleen and liver 30 minutes after injection—as all samples of the spleen and liver lay below the reliable quantification limits of the pCMV-luc standard plot (< 1 pg of plasmid per ng of total DNA in all assayed tissues; data not shown).

### Analysis of the fate of DNA complexes

Analysis of the effect of the formulation on the extent of accumulation of intact plasmid DNA at various tissue sites revealed that the administration of neutral DSPE-PEG_2000_/LP/DNA complexes resulted in significantly higher accumulation of the intact plasmid at the spleen (2.09% of injected dose) than administration of the cationic LP/DNA complexes (1.47%) (*n* = 6, *P* < 0.05, Two-way ANOVA with Tukey's *post hoc* test) at 24 hours (**Table 1**). Furthermore, the administration of both DNA complexes resulted in significantly higher levels of the intact plasmid detected at the spleen in comparison to the administration of unformulated naked plasmid (0.1%) (*n* = 5–6, *P* < 0.0001). A small percentage of the dose (~0.59%) was detected in the liver of mice injected with naked DNA, whereas, the target plasmid detected in the livers of the mice injected with LP/DNA or DSPE-PEG_2000_/LP/DNA (*n* = 6 in each case) was less than the reliable quantification limits of the assay. No significant differences were observed between the levels of target plasmid remaining at the injected calf muscle or draining popliteal lymph node after administration of either DNA complexes or unformulated plasmid.

Comparison of the tissue distribution of each administered DNA formulation, made possible via the utilization of our tissue-specific qPCR method, revealed that both the LP/DNA and DSPE-PEG_2000_/LP/DNA complexes exhibited different tissue distribution profiles to that of naked DNA 24 hours after intramuscular administration (**Figure 3b**). Overall, the spleen contained the highest mass of intact plasmid after administration of either the LP/DNA complex (*n* = 5 to 6, *P* < 0.001 compared to muscle, *P* < 0.0001 compared to all other tissues) and DSPE-PEG_2000_/LP/DNA (*P* < 0.0001 compared to other tissues). No significant differences were observed between the amounts detected at the other tissue sites after administration of either of the DNA complexes. The administration of naked DNA resulted in a residue of intact plasmid detected at all assayed tissues with the liver accumulating the highest levels of the plasmid (≤ 0.59% of injected dose at each tissue site). However, the mass of intact plasmid detected in the liver was only significantly higher than that of the draining popliteal lymph node (*P* < 0.05, *n* = 6), with the mass detected in the other assayed tissues not significantly different to one another.

## Discussion

In order to determine the absolute mass of DNA in tissue using qPCR, a standard curve constructed from an appropriate reference standard is required. Current qPCR strategies for quantifying plasmid DNA use standard curves determined from serial dilutions of known amounts of the purified target plasmid plotted against the PCR Cq value.^7,9,28,29^ Such an approach does not allow adequate estimation of the mass of plasmid in the entire tissue sample as it is not known how much total DNA can be extracted from the unit mass of each tissue. To overcome this limitation, we added known amounts of reference plasmid to pre-weighed samples of excised tissues of interest, extracted the total DNA from each sample and then carried out qPCR. This allowed us to determine a standard curve for each tissue, allowing the mass of DNA detected to be expressed as a function of mass of each tissue.

Another advantage of the method developed in this study is that the variability associated with the tissue weighing, DNA extraction or amplification of the plasmid in each tissue is common to all experimental samples in that tissue, whether test samples or standard samples. This is of particular importance as it is well known that the presence of the genomic DNA matrix can lower the amplification efficiency in qPCR. Indeed, Fu and colleagues^11^ reported that > 100 ng of gDNA in the reaction can hinder the amplification of the target template. These authors suggested that using purified plasmid DNA as a reference standard would be inappropriate when comparing it to plasmid DNA that has been extracted from tissue along with tissue gDNA. The assay used in this study ensured that both the standard reference and test samples undergo the same extraction and amplification process, and thus any inhibitory effect of gDNA is common to each qPCR reaction.

Results of the assay validation indicated high specificity and reproducibility of the qPCR method with all tissues investigated. The amplification efficiency was generally lower than would be expected for pure samples, with an amplification factor between 1.6 and 1.7 for each cycle instead of the ideal factor of 2. This suggests either non-optimal qPCR conditions or trace amounts of reaction inhibitors were present in the qPCR. Given that the amplification efficiency of purified pCMV-luc standards was higher (~81%, amplification factor of ~1.8; data not shown), this suggests that the reaction did contain inhibiting components, most likely contaminant(s) from the DNAzol homogenising solution used in the DNA extraction process. Although the use of DNAzol in conjunction with a commercial silica-based column for total DNA extraction from tissue was adapted from Jeong and colleagues,^5^ we found that using this method yielded DNA samples that had low 260/230 absorbance ratios (< 2.0)—which suggested the presence of contaminants such as phenol or ethylenediaminetetraacetic acid (EDTA) in the samples. This cannot be confirmed as, being a propriety product, the composition of DNAzol® is not disclosed. We considered using a second DNA purification procedure, such as ethanol precipitation (with chloroform-isoamyl alcohol extraction to remove trace amounts of phenol). We did not adopt this approach because it is likely to increase the variability in the overall DNA extraction procedure and thus, reduce the overall reproducibility of the assay. We used the DNA in unmodified form for the qPCR reactions, accepting the low amplification efficiency. We considered that low amplification efficiencies in qPCRs are acceptable as long as the efficiency is consistent across all samples. An adequate level of consistency was reflected in the extent of linearity of standard plot constructed, which reflects whether the reaction efficiency is the same for all samples in the assayed range. For the tissues assayed in this study, high *R*^2^ values (≥ 0.97) of the calibration curves were obtained, indicating adequate linearity within the assay range.

For any qPCR assay utilized in nonviral gene delivery biodistribution studies, there is a possibility that the material components of the complexes could interfere with the qPCR analysis due to incomplete dissociation of DNA from the other materials (*i.e.*, in this study the lipopeptide and/or PE-PEG). This could limit the amount of plasmid available in the sample for amplification, and thus, result in an underestimate of the mass of plasmid in the tissue. This is of particular concern when utilizing cationic materials (such as the cationic lipopeptides used in this study) that bind strongly to the negatively charged plasmid. To investigate this we measured the levels of plasmid recovered in tissues spiked with known amounts of naked plasmid or spiked with the same mass of DNA present as cationic LP/DNA complexes. There were no significant differences between the total mass of plasmids recovered for each formulation, indicating that the DNA condensing lipopeptide did not interfere with the qPCR assay (**Supplementary Data: Figure S1**). The assay is able to discriminate between differences of approximately twofold but the assay does not allow for greater precision. This is in part the result of establishing standard plots using samples that are themselves extracted from each tissue. Inspection of the standard plots indicates that the spiked tissues give Cq values typically within one to two cycles of each other.

The tissue-specific qPCR assay suggested that both the spleen and liver contained higher amounts of plasmid than the draining lymph node 30 minutes after intramuscular injection of naked DNA (though these values were not statistically different), whilst the conventional qPCR method suggested that lower amounts (< quantification limit of the method) of the plasmid were present per ng of total DNA extracted from the spleen and liver. These results provide evidence that the mass of target plasmid detected per ng of total DNA extracted from the tissue does not give an adequate impression of the mass of target plasmid deposited in the whole tissue. Other plasmid distribution studies using these conventional qPCR methods, such as Liu and colleagues^8^ or Ruzila and colleagues,^10^ have drawn conclusions on the distribution patterns of their injected plasmid that may not be accurate, and our studies suggest that data needs to be interpreted with caution, particularly when large organs or tissues are under consideration. We suggest that the qPCR method adopted in our study is a better approach, as misleading *in vivo* distribution data is likely to result in inadequate understanding of the structure-distribution–activity relationships of nonviral gene delivery systems.

Overall, the assay illustrated that the two DNA complexes had similar tissue distribution patterns—different to that of naked plasmid. For tissues other than muscle, higher masses of plasmid were detected in the spleen of mice injected with the DNA complexes than other tissues. The significance of this increased splenic accumulation is not known at this stage, as it is not known if the plasmid passively distributed to this site or was taken up from blood by cells of the mononuclear phagocyte system (MPS). Another possibility is that the particles could have been taken up by immune cells, macrophages or dendritic cells, either from muscle or the draining lymph node, and were then delivered to the spleen by cell migration. Further fluorescence-activated cell sorting experiments in which fluorescently labeled plasmid DNA is administered may assist in elucidating whether the plasmid is intracellularly or extracellularly located.

Although not significant, results of the tissue-calibrated qPCR assay indicated that mice injected with the cationic LP/DNA complexes retained a larger mass of plasmid in the calf muscle than the PEGylated complexes or naked plasmid at 24 hours post-injection. This is congruent with current findings illustrating that cationic DNA complexes are restricted in movement from the site of injection upon administration *in vivo*^1,27^—hypothesized to be due to the presence of abundant anionic species (e.g. proteoglycans) in the extracellular matrix that may strongly interact with these complexes and hinder their mobility in the tissue.^30,31,32^ Whether or not this property is desired is dependent on the context of the DNA delivery system—as it has been suggested that a depot formation may be advantageous in inducing sustained expression of antigens in non-viral DNA vaccines,^1,4,33^ while others have showed that reducing the cationic surface charge can improve the distribution, and thereby promote transfection, to cells other than those found at the injection site (*i.e.*, transfection of both APCs and local dermal cells).^2^

It was also noted that PEGylation of the cationic lipopeptide DNA complex did not result in higher masses of plasmid in the popliteal lymph node after 24 hours. This was inconsistent to what had been reported by Zhuang *et al.* and Carstens *et al.*,^1,3^ who found that the introduction of a PEG coating on cationic liposomes, using DSPE-PEG_2000_, resulted in enhanced lymphatic draining of particles from the site of injection. The differences in findings may be due to the fact that the latter studies carried out subcutaneous administration, whereas in this study we injected intramuscularly. Another possibility is at the 24-hour time point the PEGylated particles had already drained through the lymph nodes into blood. Calculation of the total dose accumulated in the blood would be useful as it could help to establish whether the majority of the dose is distributed systemically in an intact form or present in muscle in a degraded form at specific time points. However, the qPCR assay used here focused on estimating the total mass of plasmid in each tissue. In practice, this is achievable because each tissue can be easily excised and weighed. To evaluate the total dose accumulated in the blood, on the other hand, would first require a method to completely and consistently isolate total blood from mice and then to set up a separate blood-calibrated qPCR assay, which was not attempted in this study.

As PEGylated particles typically exhibit stealth properties, it was originally hypothesized that the DSPE-PEG_2000_/LP/DNA complexes would show reduced hepatic accumulation as the liver is one of the major sites whereby opsonized nanoparticles from the bloodstream are sequestered. However, our findings suggest that formulation of the plasmid with either nonviral DNA carriers reduced the accumulation of intact plasmid in the liver, in comparison to the plasmid levels detected after administration of the naked plasmid. Published studies using intravenous injection of radiolabeled DNA reported that approximately 30–40% of injected radioactivity could be detected in the liver of mice 24 hour after injection of PEGylated particles,^34,35^ suggesting that the fate of DNA after intramuscular injection is quite different. A possible reason for the differences observed in the intravenous studies and this study is that the DNA delivered in our complexes may have been already degraded in the liver 24 hours postinjection. Indeed as mentioned previously, qPCR is designed to reflect levels of intact plasmid, whilst radiolabelling assays used in the aforementioned studies do not. The data suggest that the previously published studies^34,35^ may have been observing the accumulation of a cleaved signal (*i.e.*, degraded product) at the 24-hour time point.

Although the qPCR method used here is useful in determining the absolute amount of intact plasmid distributed to different organs and tissues, it is more labor-intensive and less high-throughput than the quantification of the plasmid *in vivo* using conventional qPCR methods. The determination of the optimal and valid assay range for each tissue can be a slow and laborious process, particularly for studies that wish to explore the biodistribution of the plasmid to a large number of organs *in vivo*. Thus, if the purpose of a study is simply to compare the effect of different formulations on the amount of plasmid accumulated at a single tissue site, then the utilization of the conventional qPCR method is recommended. However, if the aim of a study is to compare the extent of the accumulation of intact plasmid to different tissues *in vivo*, then the tissue-specific qPCR method is recommended as it is more likely to provide an accurate representation of the plasmid distribution pattern.

In this paper, we have established a tissue-specific assay using real-time qPCR for determining the absolute mass of intact plasmid present in different tissues. The novel aspect of the approach used in this study was to account for the differences in the total genomic DNA extracted from various tissue types by constructing standard curves based on excised tissue with standard plasmid amounts added. This assay is recommended in DNA vaccine or gene therapy studies to investigate the effect of different formulations or routes of administration on plasmid distribution to a range of tissues *in vivo*.

## Materials and Methods

*Plasmid DNA.* The DNA administered in the qPCR experiments, referred to as pCMV-luc, was constructed by ligating the firefly luciferase cDNA from pGL2 basic (Promega, Fitchburg, WI) into the multiple cloning site of pcDNA3 (Life Technologies, Carlsbad, CA). Plasmid isolation was carried out using the EndoFree Plasmid Giga Kit (Qiagen, Venlo, Netherlands). Plasmid concentration was determined by measuring the absorbance at 260 nm using a Nanodrop spectrophotometer (Thermo Fisher Scientific, Waltham, MA). Purity was assessed using the 260/280 nm ratio, which was always within the range 1.7–2.0, and agarose gel electrophoresis.

*Materials for particle formation.* The lipopeptide used in this study was stearoyl-CH_2_K_3_ custom manufactured by G.L. Biochem (Shanghai, China), where C, H, and K represent cysteine, histidine, and lysine, respectively. This is a water-soluble transfection reagent, with similar derivatives described elsewhere.^12^ 1,2-distearoyl-sn-glycero-3-phosphoethanolamine-N-(methoxy(polyethylene glycol)-2000) (DSPE-PEG_2000_) was obtained from Avanti Polar Lipids (Alabaster, AL). All materials were prepared in 4-(2-hydroxyethyl)-1-piperazineethanesulfonic acid (HEPES)-glucose buffer (15 mmol/l HEPES and 5% w/v glucose, pH = 7.4).

*Mice.* Mice used in this study were C57BL/6J male mice aged between 10–12 weeks. All intramuscular injections (31 gauge needle) were introduced into the left calf muscle using a 50 µL injection volume. All mice were anaesthetised with 1–4% titrated isoflurane gas prior to intramuscular injection. Euthanasia was carried out using gaseous CO_2_. Tissues harvested in this study (injected calf muscle, draining popliteal lymph node, spleen and liver) were snap frozen on dry ice and then stored at −80°C until further use. All animal procedures were carried out under approval of ethics application MIPS.2013.16 and MIPS.2011.20, granted by the Monash Institute of Pharmaceutical Sciences Animal Ethics Committee.

*Preparation of DNA complexes* *Calculation of molar charge ratios.* In the context of the LP/DNA complexes, the charge ratio refers to the number of positively charged amine groups (NH_3_^+^) at pH 7.4, provided by the lysine residues of the cationic LP per negatively charged phosphate group (PO_4_^-^) of DNA (where 1 phosphate group = 1 nucleotide). For example, to obtain a theoretical charge ratio of 1:1 (LP:DNA) using stearoylCH_2_K_3_ (1045.45 g/mol, 3 NH_3_^+^/molecule), 1 µg of plasmid DNA (3 nmol) was mixed with 1.06 µg of stearoylCH_2_K_3_ (1 nmol). An average mass of 330 g/mol per nucleotide of DNA was used for the calculations. In the context of the DSPE-PEG_2000_/LP/DNA complexes, the charge ratio refers to the number of negatively charged phosphate group provided by the DSPE-PEG_2000_ per positively charged amine of the lysine residues of the lipopeptide per phosphate group of the DNA, respectively. For example, to obtain a theoretical charge ratio of 1:1:1 (DSPE-PEG_2000_:LP:DNA) for DSPE-PEG_2000_ (2805.54 g/mol, PO_4_^-^/molecule) and stearoylCH_2_K_3_, 1 µg of plasmid DNA (3 nmol) was mixed with 1.06 µg of stearoylCH_2_K_3_ (1 nmol) and 8.5 µg of DSPE-PEG_2000_ (3 nmol).

*Formation of DNA complexes.* LP/DNA complexes were prepared by mixing together an equal volume of solutions of the LP and DNA in HEPES-glucose buffer at a charge ratio (+/−) of 2.5 to 1. The DSPE-PEG_2000_/LP/DNA complexes were prepared at a charge ratio (−/+/−) of 0.75 to 2 to 1 by mixing DSPE-PEG_2000_ and DNA together with an equal volume of LP in HEPES-glucose buffer. Both the LP/DNA and DSPE-PEG_2000_/LP/DNA were then incubated at room temperature for 30 minutes to allow for particle formation. To concentrate the DNA complexes down to a 50 µl volume for injection, ultrafiltration was carried out using a 3kDa Amicon Ultra-15 centrifugal unit (Merck Millipore, Billerica, MA) to retain the complexes. The DNA complexes were subjected to ultrafiltration at 5,000 *g* for ~5.5 hours or until the solution retained had reached desired volume. The percentage of recovery of the DNA complexes were determined by measuring the fluorescence of samples containing fluorescently labeled plasmid DNA post-ultrafiltration and calculating the resultant concentration based on a standard plot constructed by measuring the fluorescence of known concentrations of the labeled DNA in the LP or PEGylated complexes. The fluorophore used to label the plasmid was ChromaTide Alexa Flour 594-5-dUTP (Life Technologies, Carlsbad, CA) and was covalently labelled to the linearized plasmid using the Klenow fragment (Geneworks, Thebarton, Australia) enzyme. Fluorescent measurements were carried out using the Cary Eclipse Fluorescence Spectrophotometer (Agilent Technologies, Santa Clara, CA) at the excitation/emission wavelengths of 594/614 nm.

*Particle sizing and zeta potential measurements.* The mean particle size (Z-average), polydispersity index (PI) and zeta potential (ZP) of each complex was measured using the Zetasizer Nano ZS (Malvern Instruments, Malvern, United Kingdom). All samples were prepared in triplicate and measurements were carried out at 25 °C. To ensure that the instrument was calibrated, a 60 ± 2.7 nm size standard (Malvern Instruments, Malvern, UK) and a −68 ± 6.8 mV zeta potential standard (Malvern Instruments) were measured and assessed prior to experimentation.

**Figure 4:**
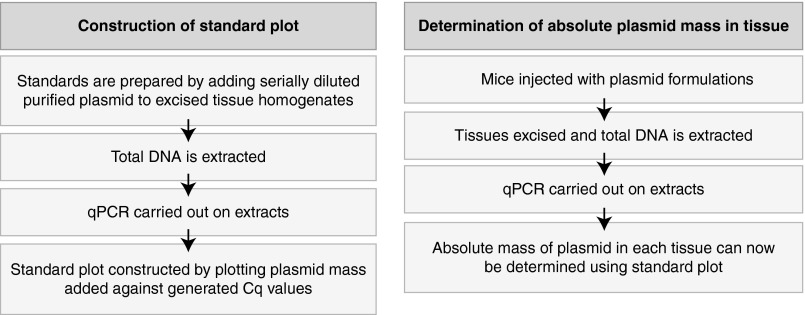
Schematic diagram providing an overview of the tissue-specific qPCR strategy.

*Quantitative PCR assay* *Overview.* An outline of the tissue-specific qPCR strategy for the quantitation of absolute plasmid levels in tissue is provided in **Figure 4**. Briefly, the method involved the construction of standard curves for each tissue assayed, by adding serially diluted plasmid standards to homogenates of each pre-weighed, excised tissue of interest. This approach allowed us to relate the amount of plasmid detected to the amount of genomic DNA extracted from each tissue, and thus, enabled the determination of the absolute amount of plasmid found at each particular site.

*Total DNA extraction.* The DNA extraction method used was adapted from Jeong and colleagues.^5^ Tissues were harvested, weighed and homogenized in DNAzol reagent (Life Technologies) using a handheld TissueTearor homogenizer (Biospec Products, Bartlesville, OK). The lymph nodes were homogenized with a Pellet Pestles motor grinder (Sigma-Aldrich, St Louis, MO). Homogenates were incubated overnight at room temperature with the addition of proteinase K (Qiagen, Venlo, Netherlands) to a final concentration of 100 µg/ml. For both the calf muscle and lymph node, the whole tissue was used and homogenized in 1.88 and 0.4 ml of DNAzol, respectively. For the spleen and liver, 5 and 9 mg of tissue, respectively, were weighed and each homogenized in 1 ml of DNAzol. The spleen extract was then further diluted by adding 0.1 ml of the homogenate to 0.9 ml of DNAzol. The tissue extracts were then centrifuged for 10 minutes at 10,000 *g* at 4 °C. Total DNA was extracted using a Wizard DNA Prep Spin column (Promega, Fitchburg, WI) using the manufacturer's instructions.

*Quantitative PCR.* Real-time quantitative PCR was carried out using the CFX96™ Real Time PCR Detection System (Bio-Rad, Hercules, CA). Forward (5' CCTCATAAAGGCCAAGAAGG 3') and reverse (5' ACACCGGCCTTATTCCAAG 3') primers were used to amplify a 114 bp fragment of the pCMV-luc. Each PCR reaction contained 5 µl of the iQ SYBR Green Supermix (Bio-Rad), 200 nmol/l of each primer, 1 ng of template DNA and water to a total reaction volume of 10 µl. Reactions were carried out with an initial incubation at 95 °C for 3 minutes, followed by 40 cycles of denaturation at 95 °C for 10 seconds, annealing at 47 °C for 30 seconds, and extension at 72 °C for 30 seconds. All reactions were carried out in triplicate and ‘no template' controls, containing water instead of template DNA, were included in every PCR run.

*Specificity of the qPCR assay.* Melting curve analysis (55 °C to 95 °C, 0.5 °C increments per 5 seconds) was carried out with the CFX96 Real Time PCR Detection System (Bio-Rad) after each qPCR experiment to confirm product specificity. Gel electrophoresis was used in addition to verify primer specificity and amplicon size using 3% agarose.

*Reproducibility of qPCR assay.* To assess the reproducibility of the qPCR assay, the coefficient of variation (CoVar) was calculated from the Cq mean and standard deviation (SD) for each tissue (*n* = 3) spiked with 100 ng of plasmid, using the following equation.

CoVar = (SD/mean Cq) × 100%

*Construction of plasmid standard plot.* Tissues from each mouse were excised, weighed, homogenized and then total DNA was extracted following the procedure described above. Standard masses of pCMV-luc (1 ng, 10 ng, 100 ng, 1 µg) were prepared and added to the homogenate prior to centrifugation. Standard plots were generated for each tissue by plotting the mass of plasmid standard introduced into the tissue sample against the mean Cq value. To assess assay validity, linear regression was used to calculate the goodness of fit (*R*^2^) of each standard plot.

*Application of qPCR assay to investigate the biodistribution of plasmid DNA.* Mice were injected intramuscularly with 50 µg of pCMV-luc plasmid (naked DNA) or formulated DNA complexes (LP/DNA or DSPE-PEG_2000_/LP/DNA) and euthanized at 30 minutes or 24 hours postinjection. Total DNA was then extracted from each excised tissue, and real-time qPCR was carried out as outlined above. As a control, mice were injected with 50 µl of the HEPES-glucose vehicle. Using the standard plots constructed for each tissue, the amount of plasmid present in the tissue was then calculated. For comparison, analysis via the conventional qPCR method was also carried out by determining the amount of plasmid (ng) per ng of total DNA, calculated from a standard plot of varying concentrations of the purified pCMV-luc (1 ng to 1 pg, 10-fold dilutions) versus the generated Cq values.

*Statistical analysis.* Data in graphs are represented as mean ± standard error of the mean (SEM) with each data point representing *n* = 4–6 samples, unless otherwise stated. One-way ANOVA with Tukey's *post-hoc* test was used to assess significant differences in the levels of plasmid after injection of naked DNA after analysis via the tissue-specific qPCR method or the conventional qPCR method. Two-way ANOVA with Tukey's *post-hoc* test was used to assess the significant differences in the levels of plasmid at each site after administration of formulated DNA complexes, with the two factors being the DNA formulation and tissue type. A *P*-value of less than 0.05 was considered to be statistically significant.

**SUPPLEMENTARY MATERIAL** **Figure S1.** Estimation of the total mass of plasmid recovered from the calf muscle spiked with naked DNA or LP/DNA formulations.

## Figures and Tables

**Table 1:**
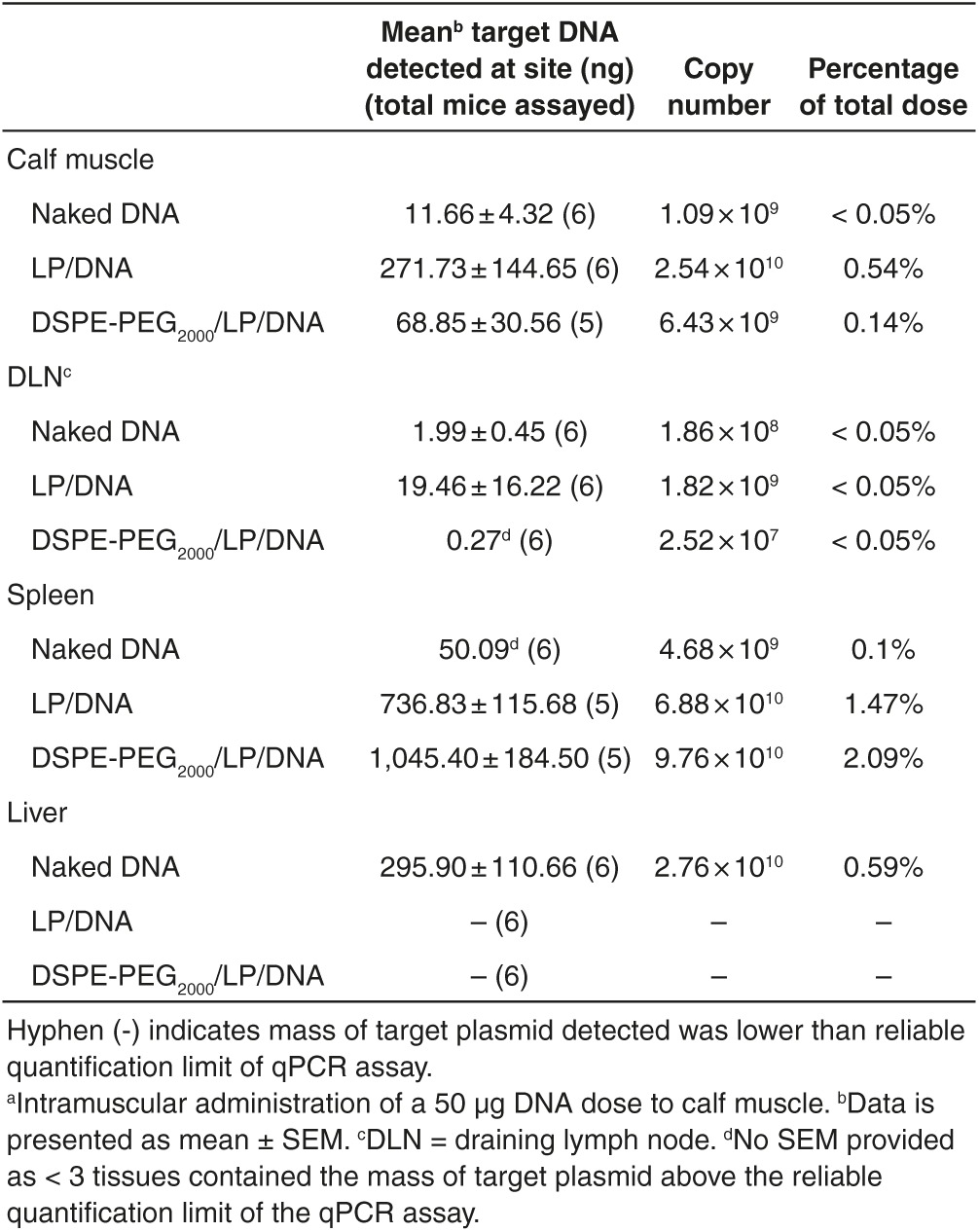
The mean mass of target DNA, copy number and percentage of total dose detected in each tissue 24 hours after injection^a^ determined using the tissue-specific qPCR method

## References

[bib1] Carstens, MG, Camps, MG, Henriksen-Lacey, M, Franken, K, Ottenhoff, TH, Perrie, Y et al. (2011). Effect of vesicle size on tissue localization and immunogenicity of liposomal DNA vaccines. Vaccine 29: 4761–4770. 21565240 10.1016/j.vaccine.2011.04.081

[bib2] Palumbo, RN, Zhong, X, Panus, D, Han, W, Ji, W and Wang, C (2012). Transgene expression and local tissue distribution of naked and polymer-condensed plasmid DNA after intradermal administration in mice. J Control Release 159: 232–239. 22300619 10.1016/j.jconrel.2012.01.012 PMC3351500

[bib3] Zhuang, Y, Ma, Y, Wang, C, Hai, L, Yan, C, Zhang, Y et al. (2012). PEGylated cationic liposomes robustly augment vaccine-induced immune responses: Role of lymphatic trafficking and biodistribution. J Control Release 159: 135–142. 22226776 10.1016/j.jconrel.2011.12.017

[bib4] Kawase, A, Kobayashi, N, Isaji, K, Nishikawa, M and Takakura, Y (2005). Manipulation of local disposition and gene expression characteristics of plasmid DNA following intramuscular administration by complexation with cationic macromolecule. Int J Pharm 293: 291–301. 15778067 10.1016/j.ijpharm.2004.12.011

[bib5] Jeong, GJ, Byun, HM, Kim, JM, Yoon, H, Choi, HG, Kim, WK et al. (2007). Biodistribution and tissue expression kinetics of plasmid DNA complexed with polyethylenimines of different molecular weight and structure. J Control Release 118: 118–125. 17250923 10.1016/j.jconrel.2006.12.009

[bib6] Khargharia, S, Kizzire, K, Ericson, MD, Baumhover, NJ and Rice, KG (2013). PEG length and chemical linkage controls polyacridine peptide DNA polyplex pharmacokinetics, biodistribution, metabolic stability and *in vivo* gene expression. J Control Release 170: 325–333. 23735574 10.1016/j.jconrel.2013.05.024 PMC3904502

[bib7] Gravier, R, Dory, D, Laurentie, M, Bougeard, S, Cariolet, R and Jestin, A (2007). *In vivo* tissue distribution and kinetics of a pseudorabies virus plasmid DNA vaccine after intramuscular injection in swine. Vaccine 25: 6930–6938. 17728026 10.1016/j.vaccine.2007.07.001

[bib8] Liu, C, Fan, M, Xu, Q and Li, Y (2008). Biodistribution and expression of targeted fusion anti-caries DNA vaccine pGJA-P/VAX in mice. J Gene Med 10: 298–305. 18085725 10.1002/jgm.1138

[bib9] Orság, P, Kvardová, V, Raska, M, Miller, AD, Ledvina, M and Turánek, J (2008). Quantitative real-time PCR study on persistence of pDNA vaccine pVax-Hsp60 TM814 in beef muscles. Genet Vaccines Ther 6: 11. 18761754 10.1186/1479-0556-6-11 PMC2542361

[bib10] Ruzila, I, Zeenathul, N, Nik-Mohd-Afizan, N, Sheikh-Omar, A, NorHidayah, M, and Mohd-Azmi, M (2010). Tissue distribution of intramuscularly and intratumouraly administered DNA plasmid harbouring apoptotic gene in mice. Afr J Pharmac Pharmacol 4: 775–782.

[bib11] Fu, J, Li, D, Xia, S, Song, H, Dong, Z, Chen, F et al. (2009). Absolute quantification of plasmid DNA by real-time PCR with genomic DNA as external standard and its application to a biodistribution study of an HIV DNA vaccine. Anal Sci 25: 675–680. 19430152 10.2116/analsci.25.675

[bib12] Tarwadi, Jazayeri, JA, Prankerd, RJ and Pouton, CW (2008). Preparation and in vitro evaluation of novel lipopeptide transfection agents for efficient gene delivery. Bioconjugate Chemistry 19: 940–950. 18333604 10.1021/bc700463q

[bib13] Ryu, DW, Kim, HA, Song, H, Kim, S and Lee, M (2011). Amphiphilic peptides with arginines and valines for the delivery of plasmid DNA. J Cell Biochem 112: 1458–1466. 21322000 10.1002/jcb.23064

[bib14] Layek, B and Singh, J (2013). Cell penetrating peptide conjugated polymeric micelles as a high performance versatile nonviral gene carrier. Biomacromolecules 14: 4071–4081. 24083483 10.1021/bm401204n

[bib15] El-Sayed, A, Masuda, T, Khalil, I, Akita, H and Harashima, H (2009). Enhanced gene expression by a novel stearylated INF7 peptide derivative through fusion independent endosomal escape. J Control Release 138: 160–167. 19465073 10.1016/j.jconrel.2009.05.018

[bib16] Adami, RC, Collard, WT, Gupta, SA, Kwok, KY, Bonadio, J and Rice, KG (1998). Stability of peptide-condensed plasmid DNA formulations. J Pharm Sci 87: 678–683. 9607943 10.1021/js9800477

[bib17] McKenzie, DL, Collard, WT and Rice, KG (1999). Comparative gene transfer efficiency of low molecular weight polylysine DNA-condensing peptides. J Pept Res 54: 311–318. 10532236 10.1034/j.1399-3011.1999.00104.x

[bib18] Plank, C, Tang, MX, Wolfe, AR and Szoka, FC Jr (1999). Branched cationic peptides for gene delivery: role of type and number of cationic residues in formation and *in vitro* activity of DNA polyplexes. Hum Gene Ther 10: 319–332. 10022556 10.1089/10430349950019101

[bib19] Chen, QR, Zhang, L, Stass, SA and Mixson, AJ (2001). Branched co-polymers of histidine and lysine are efficient carriers of plasmids. Nucleic Acids Res 29: 1334–1340. 11239000 10.1093/nar/29.6.1334 PMC29747

[bib20] Midoux, P, Kichler, A, Boutin, V, Maurizot, JC and Monsigny, M (1998). Membrane permeabilization and efficient gene transfer by a peptide containing several histidines. Bioconjug Chem 9: 260–267. 9548543 10.1021/bc9701611

[bib21] Pichon, C, Roufaï, MB, Monsigny, M and Midoux, P (2000). Histidylated oligolysines increase the transmembrane passage and the biological activity of antisense oligonucleotides. Nucleic Acids Res 28: 504–512. 10606649 10.1093/nar/28.2.504 PMC102506

[bib22] Leng, Q, Scaria, P, Ioffe, OB, Woodle, M and Mixson, AJ (2006). A branched histidine/lysine peptide, H2K4b, in complex with plasmids encoding antitumor proteins inhibits tumor xenografts. J Gene Med 8: 1407–1415. 17133339 10.1002/jgm.982

[bib23] McKenzie, DL, Kwok, KY and Rice, KG (2000). A potent new class of reductively activated peptide gene delivery agents. J Biol Chem 275: 9970–9977. 10744672 10.1074/jbc.275.14.9970

[bib24] McKenzie, DL, Smiley, E, Kwok, KY and Rice, KG (2000). Low molecular weight disulfide cross-linking peptides as nonviral gene delivery carriers. Bioconjug Chem 11: 901–909. 11087340 10.1021/bc000056i

[bib25] Kwok, KY, Park, Y, Yang, Y, McKenzie, DL, Liu, Y and Rice, KG (2003). *In vivo* gene transfer using sulfhydryl cross-linked PEG-peptide/glycopeptide DNA co-condensates. J Pharm Sci 92: 1174–1185. 12761807 10.1002/jps.10384

[bib26] van Hell, AJ, Crommelin, DJ, Hennink, WE and Mastrobattista, E (2009). Stabilization of peptide vesicles by introducing inter-peptide disulfide bonds. Pharm Res 26: 2186–2193. 19582551 10.1007/s11095-009-9933-z PMC2719749

[bib27] Chang, CW, Choi, D, Kim, WJ, Yockman, JW, Christensen, LV, Kim, YH et al. (2007). Non-ionic amphiphilic biodegradable PEG-PLGA-PEG copolymer enhances gene delivery efficiency in rat skeletal muscle. J Control Release 118: 245–253. 17270304 10.1016/j.jconrel.2006.11.025

[bib28] Tuomela, M, Malm, M, Wallen, M, Stanescu, I, Krohn, K and Peterson, P (2005). Biodistribution and general safety of a naked DNA plasmid, GTU-MultiHIV, in a rat, using a quantitative PCR method. Vaccine 23: 890–896. 15603889 10.1016/j.vaccine.2004.08.004

[bib29] Cho, HJ, Lee, S, Im, S, Kim, MG, Lee, J, Lee, HJ et al. (2012). Preclinical pharmacokinetics and biodistribution of human papillomavirus DNA vaccine delivered in human endogenous retrovirus envelope-coated baculovirus vector. Pharm Res 29: 585–593. 21948385 10.1007/s11095-011-0598-z

[bib30] Eggen, KH, Malmstrøm, A and Kolset, SO (1994). Decorin and a large dermatan sulfate proteoglycan in bovine striated muscle. Biochim Biophys Acta 1204: 287–297. 8142471 10.1016/0167-4838(94)90020-5

[bib31] Nakano, T, Sunwoo, HH, Li, X, Price, MA, and Sim, JS (1996). Study of Sulfated Glycosaminoglycans from Porcine Skeletal Muscle Epimysium Including Analysis of Iduronosyl and Glucuronosyl Residues in Galactosaminoglycan Fractions. Journal of Agricultural and Food Chemistry 44: 1424–1434.

[bib32] Ruponen, M, Ylä-Herttuala, S and Urtti, A (1999). Interactions of polymeric and liposomal gene delivery systems with extracellular glycosaminoglycans: physicochemical and transfection studies. Biochim Biophys Acta 1415: 331–341. 9889391 10.1016/s0005-2736(98)00199-0

[bib33] Endmann, A, Oswald, D, Riede, O, Talman, EG, Vos, RE, Schroff, M et al. (2014). Combination of MIDGE-Th1 DNA vaccines with the cationic lipid SAINT-18: studies on formulation, biodistribution and vector clearance. Vaccine 32: 3460–3467. 24681271 10.1016/j.vaccine.2014.03.048

[bib34] Peracchia, MT, Fattal, E, Desmaële, D, Besnard, M, Noël, JP, Gomis, JM et al. (1999). Stealth PEGylated polycyanoacrylate nanoparticles for intravenous administration and splenic targeting. J Control Release 60: 121–128. 10370176 10.1016/s0168-3659(99)00063-2

[bib35] Kommareddy, S and Amiji, M (2007). Biodistribution and pharmacokinetic analysis of long-circulating thiolated gelatin nanoparticles following systemic administration in breast cancer-bearing mice. J Pharm Sci 96: 397–407. 17075865 10.1002/jps.20813

