# Supplementary material for: Tissue-specific Calibration of Real-time PCR Facilitates Absolute Quantification of Plasmid DNA in Biodistribution Studies
Source: Mol Ther Nucleic Acids. 2016 Oct 4;5(10):e371–. doi: 10.1038/mtna.2016.79 (PMC5095683; doi:10.1038/mtna.2016.79)
Supplement: Supplementary Information [file mtna201679x1.docx]

**Supplementary Data**

**Tissue-specific calibration of real-time PCR facilitates absolute quantification of plasmid DNA in biodistribution studies**

Joan K. Ho^1^, Paul J. White^1^ Colin W. Pouton^1,2,*^

^1^ Drug Discovery Biology, Monash Institute of Pharmaceutical Sciences, Monash University (Parkville Campus), Melbourne, Australia

^2^ Drug Delivery, Disposition and Dynamics, Monash Institute of Pharmaceutical Sciences, Monash University (Parkville Campus), Melbourne, Australia

**Fig. S1. Estimation of the total mass of plasmid recovered from the calf muscle spiked with naked DNA or LP/DNA formulations.**

To assess whether the DNA condensing lipopeptide (LP) can interfere with the quantitation of the target plasmid in the qPCR assay (i.e. via the incomplete dissociation of the DNA from the cationic LP), tissues were spiked with 500ng of naked DNA or LP/DNA formulation containing 500ng DNA. Total DNA was extracted following the procedure outlined in the ‘Total DNA extraction’ methods section in the manuscript. To quantify the total plasmid recovered from the spiked tissues, qPCR was carried out following the procedure outlined in the ‘Quantitative PCR’ methods section of the manuscript, and the tissue-calibrated standard plot (plasmid mass Vs Cq) was used to calculate the total plasmid recovered. The data was obtained using four separate tissue samples (biological replicates, not technical replicates). An unpaired student’s t-test revealed that there were no significant differences in the amount of plasmid recovered from muscle spiked with the LP/DNA versus the naked DNA. This suggests that the DNA condensing lipopeptide did not interfere with the quantitation of the target plasmid in the qPCR assay. Data is represented as mean ± SEM (n =4).
